# Reference Data for Standardized Quality of Life Questionnaires in Indian Patients with Brain Metastases from Non-small Cell Lung Cancer: Results from a Prospective Study

**DOI:** 10.7759/cureus.1149

**Published:** 2017-04-10

**Authors:** Jaiprakash Aggarwal, Santam Chakraborty, Sarbani Ghosh Laskar, Vijay M Patil, Kumar Prabhash, Atanu Bhattacharya, Vanita Noronha, Nilendu C Purandare, Amit Joshi, Naveen Mummudi, Jitendra Arora, Rupali Badhe

**Affiliations:** 1 Department of Radiation Oncology, Tata Memorial Hospital, Mumbai, India; 2 Department of Medical Oncology, Tata Memorial Hospital, Mumbai, India; 3 Department of Biostatistics, Malabar Cancer Center; 4 Department of Nuclear Medicine, Tata Memorial Hospital, Mumbai, India

**Keywords:** lung cancer, health-related quality of life, brain metastases, eortc qlq c30, eortc lc13, eortc quality of life, reference data, indian experience, non-small cell lung cancers (nsclc), whole brain radiation therapy

## Abstract

**Introduction:**

Reference data for European Organization for Research and Treatment of Cancer (EORTC) quality of life questionnaires do not include studies from the Indian subcontinent. The objective of the current study was to establish a reference dataset for Indian patients of non-small cell lung cancer (NSCLC) presenting with brain metastases (BM).

**Material and methods:**

One hundred forty patients with NSCLC with BM treated between 2012-2015 were registered in a prospective cohort study (CTRI/2013/01/003299). The baseline quality of life was evaluated using the EORTC general quality of life questionnaire QLQ-C30 and lung cancer specific module LC13. Minimum important difference (MID) scores for individual domains of the EORTC QLQ-C30 and LC13 questionnaires were derived (MID = 0.2 x standard deviation) from the reference data for patients with recurrent/metastatic lung cancers. In addition, a systematic review was conducted to identify studies reporting baseline quality of life scores for recurrent/metastatic NSCLC.

**Results:**

Scores of several functional as well as symptom scales in the current NSCLC population differed by more than the MID from the baseline mean scores in the reference EORTC population as well as that reported from other studies. Differences in mean score from the EORTC reference data ranged from 6.2 and 9.4 points for the role functioning and cognitive functioning domains. In the symptom scales, the largest differences were observed for the financial difficulties (23.9) scores for the QLQ-C30 and peripheral neuropathy (21.7) for LC13 questionnaires.

**Conclusion:**

The current study demonstrates that baseline reference scores need to be established for patients from the Indian subcontinent. The findings from the current study have important implications for studies employing quality of life (QOL) assessment in the Indian NSCLC patient population.

## Introduction

As per Globocan 2012 estimates, approximately 70,000 new cases of carcinoma lung are diagnosed in India annually [1], with 75% of the patients presenting with advanced stage disease [2]. More than half of these patients receive palliative treatment [2]. Anywhere between 10-50% of these patients will present with brain metastases [3-8], and maintaining the health-related quality of life (HRQOL) is an important goal of therapy. HRQOL is a complex construct that includes the physical, psychological, social, and emotional aspects of health and includes subjective evaluation of both the positive and negative aspects [9]. HRQOL assessment has traditionally relied on the use of patient reported outcomes measured using defined questionnaires. The European Organization for Research and Treatment of Cancer (EORTC) has developed a series of modular questionnaires to evaluate the HRQOL in patients with cancers enrolled in clinical trials [9-10]. The EORTC quality of life questionnaire (QLQ-C30) is a multidimensional questionnaire of 30 questions evaluating 15 different domains affecting the HRQOL [10]. The EORTC LC13 is an add-on questionnaire that evaluates 11 symptoms domains specifically for lung cancer patients [11]. Several of these questionnaires have been validated and translated for use in the Indian population.

The EORTC reference values for their quality of life (QOL) questionnaires are derived from EORTC Quality of Life Groups’ Cross-Cultural Analysis Project data as well as individuals and organizations from non-EORTC institutes. The data is published in the EORTC QLQ-C30 reference values manual, which can be freely obtained from the EORTC QOL website (http://groups.eortc.be/qol/manuals) [12].The reference values are derived from the baseline pretreatment QOL of scores for the patients, and the manual presents the reference data for all patients as well as patients belonging to specific cancer subsites and for gender-, age-, and cancer stage-wise subgroups for each of these cancer subsites. While this reference data is derived from a large population of patients (> 23,000), patients from Asia account for only 11% of this population. Unfortunately, patients from India are completely unrepresented in this sample.

Reference data is important in QOL research as it allows comparisons with patients in different settings as well as receiving different treatments [12]. It also allows us to understand the score of an individual patient with respect to that of the general population and potentially allow us to focus interventions for improving specific symptoms or functional impairments 12]. Practically, however, the greatest utility of reference data is for calculation of sample sizes for trials in which QOL is an outcome measure.

Given the significant impact of demographic factors like age and gender on the reference values [13], it would be remiss to assume that the reference values for patients from India would be similar to that obtained from the West. For example in the study reported by Dubashi, et al. [14], the mean baseline scores for role functioning, emotional functioning, cognitive functioning, and social functioning were almost different by 10 points when compared against the reference values for breast cancer patients of similar age group as obtained from the EORTC reference value manual [12]. Similarly, mean baseline QOL values reported from Asian patients with lung cancer is often different by 10 points or more for several domains especially for social and emotional domain scores [15-17]. Thus there is a need to define the reference values for the EORTC quality of life questionnaires in Indian patients in order to facilitate the use of these questionnaires in research.

Between June 2012 to April 2015, we enrolled consecutive patients with non-small cell lung cancers (NSCLC) treated for brain metastases (BM) in a prospective registry study. The baseline QOL was measured in all these patients using the EORTC QLQ-C30 and LC13. The objective of the present paper is to present the baseline pre-treatment quality of life data for these patients so as to establish a reference dataset for this population and to compare this data with the reference data available for recurrent/metastatic lung cancer patients in the EORTC manual. This is important particularly as baseline QOL data for this population of Indian patients has not been published before and is not available in the EORTC dataset.

## Materials and methods

Consecutive patients with NSCLC presenting with BM in a tertiary care cancer center in the public sector were enrolled in a prospective observational study titled “Prospective assessment of the quality of life in patients diagnosed with carcinoma lung with brain metastases receiving short course palliative whole brain radiotherapy” (CTRI Number: CTRI/2013/01/003299). All patients had histopathological documentation of non-small cell lung cancer at the time of diagnosis and provided written informed consent prior to participation in the study. After a multidisciplinary joint clinic discussion, these patients underwent palliative whole brain radiotherapy to a dose of 20 Gy in five fractions. Whole brain radiotherapy was delivered on megavoltage equipment, once a day with bilateral parallel opposed fields. German helmet technique field shaping was done with blocks or multileaf collimators [18]. Further systemic therapy with chemotherapy or targeted therapy was given as per standard institutional protocols.

Patients underwent baseline assessment of the quality of life using the EORTC QLQ-C30, EORTC LC13, and EORTC BN20 questionnaires in addition to assessment of the Mini-Mental status prior to initiation of treatment and at three monthly intervals subsequently. The baseline Karnofsky performance status (KPS) and the risk category as per the recursive partioning analysis (RPA) was also documented. Patients received steroids, anticonvulsants, and other medications as clinically indicated in addition to chemotherapy / targeted therapy for their primary disease. Patients were followed-up until their death. In the current study, we have chosen to focus on the baseline data of the EORTC QLQ-C30 and the LC13 questionnaires only as reference data for the EORTC BN20 is not available in the EORTC Reference Values Manual [12]. The quality of life questionnaires were self-administered and patients were provided with validated vernacular translations of Hindi and Marathi versions of the EORTC provided questionnaires.

The individual items of the EORTC QLQ-C30 and LC13 questionnaires were summed and transformed to generate domain scores as per the methodology provided in the EORTC QLQ Scoring Manual [19]. Briefly, the raw score was calculated as the mean of the values for all of the components in a given scale. The raw score was then transformed linearly to standardize it by dividing it by the range of the items in the scale. Scores for functional scales were reverse coded so that higher scores indicated a higher functional status, while a higher score in a symptom scale indicated a greater symptomatic burden.

The baseline scores for each functional and symptom scale was then tabulated against the reference values provided in the EORTC Reference Values Manual. As the patients in the current study had metastatic disease, we decided to contrast the scores against the reference values of lung cancer patients with recurrent/metastatic disease. As the distribution of domain/symptom scale scores are highly skewed and individual patient data was not available from the reference values manual we decided not to apply statistical tests for comparing the difference in values central tendencies or perform an agreement analysis. Instead, we checked if the difference in the mean score in the current popular was more than the minimal important differences (MID)  of the EORTC QLQ-C30 scale scores. The MID value was taken as 0.2 of the standard deviation as recommended by Samsa, et al. [20]. In addition, Maringwa, et al. also showed that anchor based (one point decrease in PS or weight loss < 20% of baseline) MID values for deterioration were closer to 0.2 SD estimates [21].

In addition, we also conducted a systematic review to compare the baseline scores in the current study against the baseline scores of patients with recurrent/metastatic lung cancer reported earlier in other studies. The methodology of the review in the current study is outlined in Appendix A and the results of the same are available in Appendix B (available online at the following URL: https://docs.google.com/spreadsheets/d/1bOY0YQ5Dd1AC3LO7f-CWxus768xkf3MiM2AtGKtE9q4/edit?usp=sharing).

The mean scores for each domain reported in these studies were extracted into a spreadsheet (Appendix B). The grand weighted mean for these mean scores (weighted for the sample size) was then computed using the SDMTools package (version 1.1) in R (version 3.2.4) (R Core Team, Vienna, Austria) [22]. We used the grand weighted mean (GWM) instead of the grand mean as there was significant variation in the sample size of these studies and mean scores for all domains were not reported by all studies. The use of the weighted mean ensured that a more representative population mean would be obtained as studies with a larger population would receive a proportionately higher weight. In addition, the standard deviation of this GWM was also calculated. Wherever reported, the scores of the different groups in the same study were used separately for the calculation of the weighted mean.

## Results

### Patient characteristics

The patient characteristics of the 140 patients of the current study are compared against the sample used for deriving the reference values for patients with recurrent/metastatic lung cancer (Table 1). As can be seen, the patient population in the current study is much younger with a mean age of 53.3 (25-75) years, and none of the patients had a small cell histology or mesothelioma.

**Table 1:** Characteristics of the current study sample compared against the EORTC QLQ-C30 recurrent / metastatic lung cancer sample. Gender was unknown for four percent patients in the EORTC cohort. NSCLC = Non Small Cell Lung Cancer, SCLC = Small Cell Lung Cancer.

	Groups	Current Study (N = 140)	EORTC (N = 307)
Age	< 40	12 (9%)	4 (1%)
	40 - 49	33 (24%)	40 (13%)
	50 - 59	56 (40%)	76 (25%)
	60 - 69	30 (21%)	110 (36%)
	70 - 79	9 (6%)	68 (22%)
	80+	0 (0%)	9 (3%)
Gender	Male	85 (61%)	197 (64%)
	Female	55 (39%)	98 (32%)
Pathology	NSCLC	140 (100%)	11 (4%)
	SCLC	NA	115 (37%)
	Mesothelioma	NA	6 (2%)
	Unknown	NA	175 (57%)

Other socio-demographic characteristics of the patient population in the current study are presented in Table 2. The median duration between the diagnosis and detection of brain metastases was 12.5 days (IQR: 0 - 281 days).

**Table 2:** Baseline characteristics of the patient population. KPS = Karnofsky Performance Status, RPA = Recursive Partitioning Analysis. Note that data regarding these characteristics are not available in the EORTC reference values manual.

Variable	Category	Frequency (%)
KPS	< 70	39 (27.9%)
	70 - 80	86 (61.4%)
	90	15 (10.7%)
Comorbidities	Yes	28 (20%)
	No	112 (80%)
Pathology	Adenocarcinoma	135 (96.4%)
	Non-adenocarcinoma	5 (3.6%)
Symptomatic Brain Metastases	Yes	127 (90.7)
	No	13 (9.3%)
Higher Mental Functions	Not affected	126 (90%)
	Affected	14 (10%)
Motor Deficits	Yes	26 (18.6%)
	No	114 (81.4%)
Cranial Nerve Palsy	Yes	13 (9.3%)
	No	127 (90.7%)
Number of Brain Metastases	1	6 (4.3%)
	2	25 (17.9%)
	3	12 (8.6%)
	4 or more	97 (69.3%)
RPA Score	I	8 (5.7%)
	II	96 (68.6%)
	III	36 (25.7%)

### Global QOL and functional domain scores

The mean scores for the five functional domains in the EORTC QLQ-C30 ranged from 57.05 for the physical functioning domain to 66.55 for social domain. Table 3 shows the differences in the functional domain scores between the present population and EORTC reference population. A difference exceeding the MID was observed for the cognitive and emotional functioning domains as compared to the mean scores in the EORTC reference data as well as the GWM scores derived from other studies.

**Table 3:** Table showing EORTC QLQ-C30 functional domain scores in the current study versus the reference values and the values obtained from systematic review of other studies. MID = Minimal Important Difference for each domain rounded to 1 digit, SD = Standard Deviation, GWM = Grand Weighted Mean. ♰ = Difference greater than MID as compared to mean score from the EORTC reference dataset, * = Difference greater than MID as compared to weighted mean from other studies. Note that reference values for physical functioning score not available in the Reference Values manual for recurrent / metastatic lung cancer patients.

Domain	MID	Present Study	EORTC	Other Studies
		Mean ± SD	Mean ± SD	GWM ± SD of GWM
Global QOL*	6	42.9 ± 29.0	43.5 ± 28.2	55.0 ± 5.1
Physical Functioning	-	57.5 ± 27.8	NA	66.4 ± 7.9
Role Functioning♰	7	61.31 ± 34.6	47.0 ± 33.9	58.9 ± 9.4
Emotional Functioning♰*	5	58.5 ± 28.6	64.7 ± 25.7	68.8 ± 4.6
Cognitive Functioning♰*	5	65.2 ± 31.0	74.6 ± 26.6	81.8 ± 5.2
Social Functioning	6	66.6 ± 33.8	64.6 ± 31.4	70.7 ± 7.3

### QLQ-C30 symptom scales

The difference in the symptom scales scores in between the current and the reference dataset is shown in Table 4. The mean symptom scores in the current population ranged from 10.7-53.7. A difference exceeding the MID was observed for nausea/vomiting, dyspnea and appetite loss symptom scale scores as compared to the mean scores in the EORTC reference data as well as the GWM scores derived from other studies. Also noteworthy is the higher financial distress score in the current population, which was almost double as compared to the EORTC reference population and that of the GWM observed in other studies.

**Table 4:** Table showing the EORTC QLQ-C30 symptom scales' scores in the current study versus the reference values and the values obtained from systematic review of other studies. SD = Standard Deviation. MID = Minimal Important Differences calculated using the standard deviation of the EORTC reference dataset. GWM = Grand Weighted Mean. ♰ = Difference greater than MID as compared to mean score from the EORTC reference dataset, * = Difference greater than MID as compared to weighted mean from other studies

Domain	MID	Present Study	EORTC	Other Studies
		Mean ± SD	Mean ± SD	GWM ± SD of GWM
Fatigue*	6	53.7 ± 27.9	51.6 ± 28.7	42.5 ± 8.1
Nausea Vomiting♰*	5	30.0 ± 28.6	16.1 ± 26.2	10.5 ± 4.1
Pain*	7	46.9 ± 31.6	40.5 ± 33.5	30.7 ± 5.2
Dyspnea♰*	7	30.0 ± 31.0	42.8 ± 34.3	39.1 ± 11.1
Insomnia	7	30.0 ± 31.5	36.4 ± 32.8	31.0 ± 5.7
Appetite Loss♰*	7	47.4 ± 36.2	34.7 ± 37.0	29.0 ± 8.9
Constipation♰	7	23.8 ± 30.5	34.0 ± 36.5	22.2 ± 6.1
Diarrhea	5	10.7 ± 22.7	12.4 ± 23.4	6.9 ± 2.7
Financial Difficulties♰*	6	46.2 ± 38.9	22.3 ± 31.3	16.7 ± 9.1

### LC13 symptom scales

Table 5 shows the scores for the symptom scales in the EORTC LC13 questionnaires for these patients. A difference exceeding the MID was observed for several symptom scale scores (sore mouth, dysphagia, alopecia, peripheral neuropathy and pain in the arm, shoulders as well as other parts of the body) as compared to the mean scores in the EORTC reference data as well as the GWM scores derived from other studies. The higher scores for sore mouth, dysphagia, peripheral neuropathy and alopecia may be indicative of the symptomatic burden arising out of the greater use of systemic chemotherapy in the current population.

**Table 5:** Table showing the EORTC LC13 symptom scales scores in the current study versus the reference values and the values obtained from systematic review of the other studies. SD = Standard Deviation, MID = Minimal Important Differences calculated using the standard deviation of the EORTC reference dataset. GWM = Grand Weighted Mean.♰ = Difference greater than MID as compared to mean score from the EORTC reference dataset, * = Difference greater than MID as compared to weighted mean from other studies.

Domain	MID	Present Study	EORTC	Other Studies
		Mean ± SD	Mean ± SD	GWM ± SD of GWM
Dyspnea	6	34.4 ± 28.3	38.3 ± 25.0	36.9 ± 8.1
Cough	5	32.9 ± 32.7	36.3 ± 23.9	38.8 ± 7.7
Hemoptysis	4	6.4 ± 18.3	7.4 ± 17.7	6.6 ± 4.3
Sore Mouth♰*	3	20.0 ± 29.1	4.8 ± 13.4	6.1 ± 2.3
Dysphagia♰*	3	18.6 ± 29.5	7.4 ± 15.3	7.8 ± 2.2
Peripheral Neuropathy♰*	4	30.7 ± 31.5	9.0 ± 19.6	13.7 ± 5.4
Alopecia♰*	3	20.2 ± 33.9	2.7 ± 12.8	11.7 ± 7.3
Pain Chest*	5	30.2 ± 33.2	25.6 ± 24.9	20.6 ± 5.2
Pain Arm / Shoulders♰*	6	30.5 ± 34.3	20.5 ± 28.7	22.5 ± 4.0
Pain Other Parts♰*	6	36.2 ± 38.0	25.4 ± 29.4	27.3 ± 5.6

## Discussion

As the current study indicates there are differences in the scores for several domains in both EORTC QLQ-C30 and LC13 among the current population. Worse cognitive function scores in the current population are possibly a consequence of the current patient population having brain metastases. The higher symptomatic burden in the current population is reflected in the higher scores for several symptom scales in both questionnaires. In any case, the lower scores of these scales in the EORTC reference population does indicate that despite having a recurrent / metastatic disease, the patient population may not reflect the baseline QOL scores in patients with brain metastases from lung cancer in general. The high frequency of small cell cancer in the EORTC reference population may be one of the factors related to this discrepancy. However, a similar pattern of difference was noted in the scores when the current population and the weighted average scores obtained from other studies were compared.

The different age profile in the current population as well as the higher financial difficulty score point may be a consequence of the socio-economic and demographic characteristics of the Indian population. Lack of universal health care and medical insurance coverage are the reasons for the higher financial burdens experienced by our patient population. In this respect, the data are similar to that reported from a rural cancer center in India where financial difficulties were a cause of distress in approximately 50% of the patient population [23]. As Mahal, et al. have shown, a large proportion of families with cancer have a significant burden of out-of-pocket expenditures and loans for the treatment of cancer [24].

Despite the differences in scores in several domains, the overall global HRQOL did not differ much between the current and reference population, which may be an indicator that the patients do perceive their quality of life not only as a function of their disease status and symptomatic burden. This is an indirect indicator of the robustness of this measure as well as a testament to the validity of the EORTC QLQ questionnaire for quality of life assessment in cancer patients.

To our knowledge, this current study is the first of its kind reported from the Indian subcontinent that has systematically evaluated the quality of life in consecutive patients with lung cancer with brain metastases. The strengths of the study include the prospective nature of the study and the emphasis on complete data collection. The homogenous patient population, with a well-defined histology and treatment received prior to bone metastases, are other important strengths. This is reflected in the small standard deviations and interquartile ranges for most of the symptom scales. However, whether these scores are representative of the scores for other recurrent/metastatic lung cancer patients remains to be established.

## Conclusions

Reference quality of life scores for two most commonly used instruments in patients with lung cancer EORTC QLQ-C30 and LC13 are likely to be variable depending on the disease characteristics as well as the demographic nature of the population. To our knowledge, this data is the first dataset of reference values derived from an Indian population, which is not represented in the EORTC QLQ reference values manual. The results strongly support the need to conduct prospective studies in a large well-defined patient population in our country to establish reference value ranges for the EORTC quality of life questionnaires.

## Appendices

### Appendix A

Reference data for EORTC QLQ-C30 and LC13 questionnaires in Indian patients with brain metastases from non-small cell lung cancer: results from a prospective study A search for relevant quality of life literature reporting the baseline quality of life was done through PubMed. The search strategy employed the following keywords as illustrated in the table below (Table 6). The advanced search feature available in PubMed was then used to join the results from the individual searches to generate the final list of abstracts for selection. Further filters for the abstracts were not employed.

**Table 6:** Pubmed search strategy

Search(((((((((((Lung Cancer) OR Non small cell lung cancer) OR small cell lung cancer) OR NSCLC) OR SCL) OR Squamous cell carcinoma lung) OR adenocarcinoma lung) OR carcinoma lung) OR pulmonary neoplasia) OR oat cell lung cancer[MeSH Terms]) OR lung carcinoma, non small cell[MeSH Terms]) OR lung cancer[MeSH Terms]Sort by:Relevance	276791
Search(((EORTC quality of life) OR EORTC QLQ C30) OR EORTC QLQ-C30) OR EORTC C30Sort by:Relevance	3039
Search((((QLQ-LC13) OR QLQ LC13) OR EORTC LC13) OR EORTC Lung Cancer Module) OR EORTC LC-13Sort by:Relevance	144
Search((((((QLQ-LC13) OR QLQ LC13) OR EORTC LC13) OR EORTC Lung Cancer Module) OR EORTC LC-13)) OR ((((EORTC quality of life) OR EORTC QLQ C30) OR EORTC QLQ-C30) OR EORTC C30)Sort by:Relevance	3063
Search(((((((((((((Lung Cancer) OR Non small cell lung cancer) OR small cell lung cancer) OR NSCLC) OR SCL) OR Squamous cell carcinoma lung) OR adenocarcinoma lung) OR carcinoma lung) OR pulmonary neoplasia) OR oat cell lung cancer[MeSH Terms]) OR lung carcinoma, non small cell[MeSH Terms]) OR lung cancer[MeSH Terms])) AND (((((((QLQ-LC13) OR QLQ LC13) OR EORTC LC13) OR EORTC Lung Cancer Module) OR EORTC LC-13)) OR ((((EORTC quality of life) OR EORTC QLQ C30) OR EORTC QLQ-C30) OR EORTC C30))Sort by:Relevance	317

The final combined search resulted in 317 unique papers. These 317 papers were reviewed using the online literature review interface provide by Covidence (Melbourne, Australia) (https://www.covidence.org/) for suitability for full text review. Abstracts of studies dealing with advanced / metastatic lung cancer where at least one or more quality of life instruments (EORTC QLQ-C30 / LC13) had been used were then selected for full text review by a single author (SC). This yielded 66 studies for further full text review.

Subsequently a full text review was undertaken on the Covidence website only after full text articles were retrieved from the Tata Medical Center (TMC) library access. These full texts were then jointly reviewed by two authors (SC and JP). Selection criteria included only those studies where a significant proportion of patients were recurrent / metastatic NSCLC and at least two or more baseline domain scores for EORTC QLQ-C30 / LC13 was reported. In studies where only plots were presented, the score was read off from the Y axis manually. Studies reporting differences or changes in the domain level scores in the follow-up were not included. Studies for which full text could not be retrieved were also excluded. A total of 18 studies were retained at this step. Separate quality scoring of these studies was not done as we wanted to include the scores where available given the limited number of studies that were found. The list of 18 studies finally included in the review are shown below.

Data of 4808 patients included in these 18 patients were included in the analysis. The weighted mean age was 61.7 years. The proportion of patients with stage IV/recurrent disease ranged between 22-100%. The weighted mean of the proportion of patients with stage IV/recurrent disease was 73%. The weighted mean of the proportion of patients with adenocarcinoma histology was 48.4%.

The details of the individual domain scores recorded for the patient population is available in Appendix B.

### List of studies included in the review

1. Bezjak A, Tu D, Seymour L, Clark G, Trajkovic A, Zukin M, et al. Symptom improvement in lung cancer patients treated with erlotinib: quality of life analysis of the National Cancer Institute of Canada Clinical Trials Group Study BR.21. J Clin Oncol 2006;24:3831–7.

2. Blackhall F, Kim D-W, Besse B, Nokihara H, Han J-Y, Wilner KD, et al. Patient-reported outcomes and quality of life in PROFILE 1007: a randomized trial of crizotinib compared with chemotherapy in previously treated patients with ALK-positive advanced non-small-cell lung cancer. J Thorac Oncol 2014;9:1625–33.

3. Cheng X, Zhou D, Lv L. Factors affecting the quality of life in lung cancer patients measured by EORTC QLQ questionnaire. Zhongguo Fei Ai Za Zhi 2004;7:230–5.

4. Gridelli C, Gallo C, Di Maio M, Barletta E, Illiano A, Maione P, et al. A randomised clinical trial of two docetaxel regimens (weekly vs 3 week) in the second-line treatment of non-small-cell lung cancer. The DISTAL 01 study. Br J Cancer 2004;91:1996–2004.

5. Grønberg BH, Sundstrøm S, Kaasa S, Bremnes RM, Fløtten Ø, Amundsen T, et al. Influence of comorbidity on survival, toxicity and health-related quality of life in patients with advanced non-small-cell lung cancer receiving platinum-doublet chemotherapy. Eur J Cancer 2010;46:2225–34.

6. Helbekkmo N, Strøm HH, Sundstrøm SH, Aasebø U, Von Plessen C, Bremnes RM, et al. Chemotherapy and quality of life in NSCLC PS 2 patients. Acta Oncol 2009;48:1019–25.

7. Helsing M, Bergman B, Thaning L, Hero U. Quality of life and survival in patients with advanced non-small cell lung cancer receiving supportive care plus chemotherapy with carboplatin and etoposide or supportive care only. A multicentre randomised phase III trial. Joint Lung Cancer Study Group. Eur J Cancer 1998;34:1036–44.

8. Kaptein AA, Yamaoka K, Snoei L, Kobayashi K, Uchida Y, van der Kloot WA, et al. Illness perceptions and quality of life in Japanese and Dutch patients with non-small-cell lung cancer. Lung Cancer 2011;72:384–90.

9. Langendijk JA, ten Velde GP, Aaronson NK, de Jong JM, Muller MJ, Wouters EF. Quality of life after palliative radiotherapy in non-small cell lung cancer: a prospective study. Int J Radiat Oncol Biol Phys 2000;47:149–55.

10. Mu X-L, Li L-Y, Zhang X-T, Wang S-L, Wang M-Z. Evaluation of safety and efficacy of gefitinib (’iressa', zd1839) as monotherapy in a series of Chinese patients with advanced non-small-cell lung cancer: experience from a compassionate-use programme. BMC Cancer 2004;4:51.

11. Smith IE, O’Brien ME, Talbot DC, Nicolson MC, Mansi JL, Hickish TF, et al. Duration of chemotherapy in advanced non-small-cell lung cancer: a randomized trial of three versus six courses of mitomycin, vinblastine, and cisplatin. J Clin Oncol 2001;19:1336–43.

12. Sundstrom S, Bremnes R, Aasebo U, Aamdal S, Hatlevoll R, Brunsvig P, et al. Hypofractionated palliative radiotherapy (17 Gy per two fractions) in advanced non-small-cell lung carcinoma is comparable to standard fractionation for symptom control and survival: A national phase III trial. J Clin Oncol 2004;22:801–10.

13. Wachters FM, Van Putten JWG, Kramer H, Erjavec Z, Eppinga P, Strijbos JH, et al. First-line gemcitabine with cisplatin or epirubicin in advanced non-small-cell lung cancer: a phase III trial. Br J Cancer 2003;89:1192–9.

14. Wintner LM, Giesinger JM, Zabernigg A, Sztankay M, Meraner V, Pall G, et al. Quality of life during chemotherapy in lung cancer patients: results across different treatment lines. Br J Cancer 2013;109:2301–8.

15. Maringwa JT, Quinten C, King M, Ringash J, Osoba D, Coens C, et al. Minimal important differences for interpreting health-related quality of life scores from the EORTC QLQ-C30 in lung cancer patients participating in randomized controlled trials. Support Care Cancer 2011;19:1753–60.

16. Brabo EP, Paschoal MEM, Biasoli I, Nogueira FE, Gomes MCB, Gomes IP, et al. Brazilian version of the QLQ-LC13 lung cancer module of the European Organization for Research and Treatment of Cancer: preliminary reliability and validity report. Qual Life Res 2006;15:1519–24.

17. Guzelant A, Goksel T, Ozkok S, Tasbakan S, Aysan T, Bottomley A. The European Organization for Research and Treatment of Cancer QLQ-C30: an examination into the cultural validity and reliability of the Turkish version of the EORTC QLQ-C30. Eur J Cancer Care 2004;13:135–44.

18. Larsson M, Ljung L, Johansson BBK. Health-related quality of life in advanced non-small cell lung cancer: correlates and comparisons to normative data. Eur J Cancer Care 2012;21:642–9.

### Appendix B

Appendix B is available online at the following URL: https://docs.google.com/spreadsheets/d/1bOY0YQ5Dd1AC3LO7f-CWxus768xkf3MiM2AtGKtE9q4/edit?usp=sharing.
